# A Moderate-Affinity Antibody–Drug Conjugate Targeting B7-H3 Exerts Potent Antitumor Efficacy

**DOI:** 10.3390/ph19040596

**Published:** 2026-04-08

**Authors:** Ziyu Zhang, Huifang Zong, Zhen Li, Shusheng Wang, Xiaodong Xiao, Yueqing Xie, Jianwei Zhu

**Affiliations:** 1 Engineering Research Center of Cell & Therapeutic Antibody, Ministry of Education, School of Pharmacy, Shanghai Jiao Tong University, Shanghai 200240, China; zhang-ziyu-0w0@sjtu.edu.cn; 2 Jecho Institute Co., Ltd., Shanghai 200240, China; zhaoxiliunian@sjtu.edu.cn (H.Z.); zhen.li@jechoinst.com (Z.L.); xiaodong.xiao@jecholabs.com (X.X.); 3 Jecho Laboratories, Inc., Frederick, MD 21704, USA; shusheng.wang@jecholabs.com

**Keywords:** antibody–drug conjugate, B7-H3, cancer therapy, solid tumor, developability

## Abstract

**Background**: B7-H3, a type I transmembrane glycoprotein belonging to the B7 superfamily, is an attractive target for antitumor therapies. B7-H3 demonstrates aberrant overexpression in various types of solid tumors while showing limited and low expression in normal human organs. Various types of treatment targeting B7-H3 have been reported. Among these treatments, antibody–drug conjugates (ADCs) have shown potent activity, and several clinical trials, including DS7300a and MGC018, are currently ongoing. **Methods**: Here, we constructed CD276-8 ADC, composed of the anti-B7-H3 antibody CD276-8 with moderate affinity, an enzymatically cleavable tetra-peptide-based linker and DXd. Characteristics, including in vitro binding affinity and internalization activity, were assessed by bio-layer interferometry (BLI), flow cytometry and high content analysis (HCA). The cytotoxicity of CD276-8 ADC was evaluated in cell lines expressing B7-H3. Pharmacokinetic profiles and antitumor activity were evaluated in mouse models in vivo. Finally, the developability of CD276-8 ADC was assessed with plasma stability, accelerated stability and freeze–thaw studies using LC-MS and HPLC. **Results**: Characterization in vitro demonstrated the moderate affinity and acceptable internalization activity of CD276-8 ADC. In addition, CD276-8 ADC exhibited potent antitumor activities in B7-H3-positive cell line-derived xenograft (CDX) models with acceptable pharmacokinetic profiles, although it showed less potent cytotoxicity in various cell lines in vitro, indicating acceptable developability. **Conclusions**: We developed CD276-8 ADC, a B7-H3-targeting ADC with moderate affinity, which delivers the TOP1 inhibitor DXd. This design combined moderate affinity and acceptable pharmacokinetics, resulting in potent antitumor efficacy in vivo. Our study suggests that affinity optimization could be a useful consideration for enhancing ADC efficacy, positioning CD276-8 ADC as a promising therapeutic for B7-H3-expressing solid tumors.

## 1. Introduction

B7-H3, also known as CD276, is a type I transmembrane glycoprotein belonging to the B7 superfamily, which includes immune checkpoints like PD-L1, B7-1 (CD80) and B7-2 (CD86) [1]. Studies have reported that the overexpression of B7-H3 in tumor tissues is correlated with poor clinical prognosis in various types of cancers. Additionally, immunohistochemistry (IHC) studies further identify its surface expression on stromal components within the tumor microenvironment (TME), involving fibroblasts, vascular endothelial cells, pericytes and transformed epithelial cells [2,3]. Clinical investigations across multi-institutional cohorts have revealed that B7-H3 demonstrates aberrant overexpression in diverse solid malignancies, including non-small cell lung cancer (NSCLC), pancreatic ductal adenocarcinoma, hepatocellular carcinoma, breast carcinoma, prostate adenocarcinoma, and cutaneous melanoma [2,4,5,6]. On the other hand, despite broad expression at the mRNA level in normal human tissues, the protein expression of B7-H3 is limited and remains at relatively low levels in normal human organs such as the liver, pancreas, ovary, kidney and prostate [2].

Recent studies suggest that B7-H3 is a coinhibitory molecule that inhibits the activation and proliferation of T-cells [1,7] and promotes pro-tumor functions such as tumor progression, migration, invasion, drug resistance, metabolism, and angiogenesis [8]. Although the physiologic functions of B7-H3 are still incompletely characterized, it is expected to be a novel and attractive pan-tumor target for antitumor therapies due to its expression profiles. Various treatment methods targeting B7-H3 include monoclonal antibodies (mAbs) with enhanced antibody-dependent cellular cytotoxicity (ADCC), T-cell engager (TCE) bispecific antibodies, chimeric antigen receptor (CAR)-T cells, and ADCs [3,9].

ADCs are composed of a mAb, linker, and biologically active payload like cytotoxic drugs. They are designed to exert potent antitumor activity against tumor cells expressing the targeted antigen by delivering the payload into the cells [10,11,12]. In total, 19 ADCs have been approved globally, and numerous clinical studies of new ADCs are ongoing [10,11]. As for B7-H3, clinical studies of several ADCs targeting B7-H3, like DS7300a, MGC018, and YL201, are ongoing [9,13]. Although several B7-H3-targeting ADCs have advanced into clinical development, none have yet received regulatory approval for any cancer indication. Clinical studies of these agents have revealed significant challenges, including safety concerns and variable efficacy across patient populations. For instance, the Phase II trial of MGC018 in prostate cancer was halted due to severe adverse events, including patient deaths [14]. Similarly, the Phase III trial of DS7300 (IDeate-Lung02) was halted due to patient deaths. These observations emphasize that there remains an urgent and unmet need for novel B7-H3 ADCs with improved safety profiles and enhanced antitumor efficacy to provide meaningful treatment options for patients with B7-H3-expressing solid tumors.

Therefore, we generated CD276-8 ADC, a moderate-affinity B7-H3-targeting ADC that is composed of a humanized anti-B7-H3 mAb and deruxtecan (mc-GGFG-DXd), a linker-payload technology from Daiichi Sankyo that has been confirmed to be stable and potent in previous clinical studies. ADCs using mc-GGFG-DXd, like trastuzumab deruxtecan targeting HER2 and datopotamab deruxtecan targeting Trop2, have been developed and approved [15,16]. Therefore, we chose mc-GGFG-DXd to prepare ADCs to evaluate the activity of antibodies. The ADC is designed to bind to B7-H3 on the cell surface and release DXd in the cytoplasm after being internalized into the cell and being cleaved in lysosomes by enzymes. The released DXd inhibits TOP1 activity and leads to the apoptosis of target cancer cells. Here, we demonstrate preclinically that CD276-8 ADC is well capable of inhibiting tumor growth or regressing tumor volume in multiple B7-H3 positive models. Furthermore, our studies, including pharmacokinetics and in vitro stability assessment of CD276-8 ADC, suggest its safety and druggability. Taken together, these studies indicate that CD276-8 ADC has the potential to be a promising ADC for solid tumor malignancies.

## 2. Results

### 2.1. Expression of B7-H3 in Cancer Cell Lines

**Figure 1:**
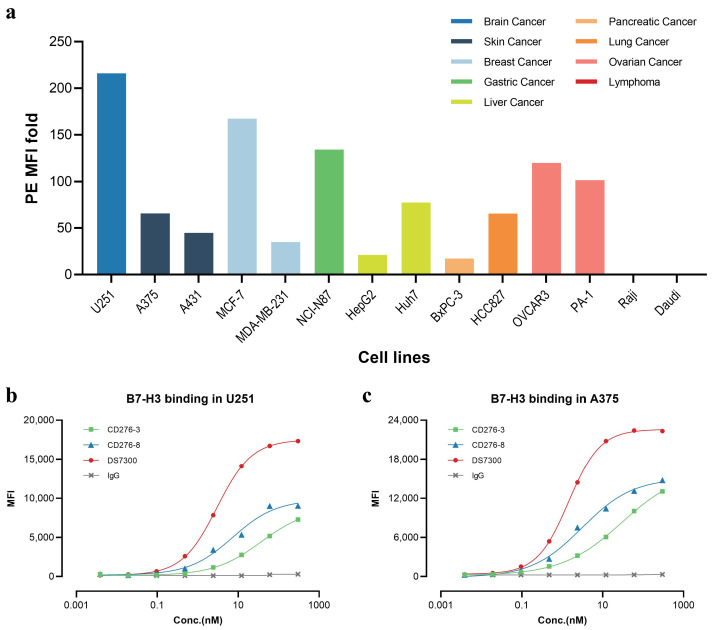
Expression of B7H3 in cell lines and in vitro binding affinity of CD276-3, CD276-8 and DS7300. (**a**) Cell surface expression of B7H3 in cell lines, as determined by FACS in the form of median fluorescence intensity (MFI) fold using R-Phycoerythrin (PE)-conjugated anti-human IgG Fc antibody. Different colors represent different types of cancer. Cell surface binding affinity of CD276-3, CD276-8 and DS7300 against B7-H3 in U251 (**b**) and A375 (**c**) cells, as determined by FACS (n = 1).

To investigate the cell surface expression of B7-H3 in cell lines, we conducted fluorescence-activated cell sorting (FACS) analysis (Figure 1a), and most solid tumor cell lines analyzed displayed high B7-H3 positive signals, showing high median fluorescence intensity (MFI) over 20 folds. In contrast, lymphoma cell lines like Raji and Daudi were determined negative. The results of FACS indicated the high expression of B7-H3 in various types of solid tumors but not in hematological cancer including leukemia, lymphoma and myeloma, consistent with datasets from the Human Protein Atlas (HPA) dataset and previous studies [2,4,5,6].

### 2.2. Characterization of Parental Antibodies CD276-3 and CD276-8

We generated CD276-3 and CD276-8 targeting human B7-H3 as candidate antibodies in order to obtain an anti-B7-H3 ADC for anti-cancer studies. The binding affinity against recombinant human B7-H3 (rhB7-H3) (4Ig) was assessed by BLI, and the K_D_ of CD276-3, CD276-8 and DS7300 were 1.24 nM, 2.12 nM and 0.231 nM (Table 1, Figure S1). The cell surface binding affinity was detected in cell lines with different B7-H3 expressions, including the B7-H3 high expression cell line U251 and the medium-level expression cell line A375 (Figure 1b,c). The affinity results were 37.32 nM and 7.246 nM for the EC_50_ of CD276-3 and CD276-8 in U251, respectively, and 29.60 nM and 3.115 nM for the EC_50_ of CD276-3 and CD276-8 in A375, respectively, while DS7300 manifested higher affinity with an EC_50_ of 2.981 nM in U251 and 1.452 nM in A375. Both candidates demonstrated moderate binding affinity with a lower span and higher EC_50_ compared with DS7300. However, the precise EC_50_ values of individual antibodies may be subject to variability due to the performance of a single experiment in our cell binding assays. Further, the epitope grouping of CD276-3, CD276-8 and DS7300 was analyzed by BLI (Figure S2a). A heatmap was generated according to the inhibition between candidates, demonstrating that CD276-3 shared a similar binding epitope to DS7300 while CD276-8 bound to a different epitope (Figure S2b).

**Figure 2:**
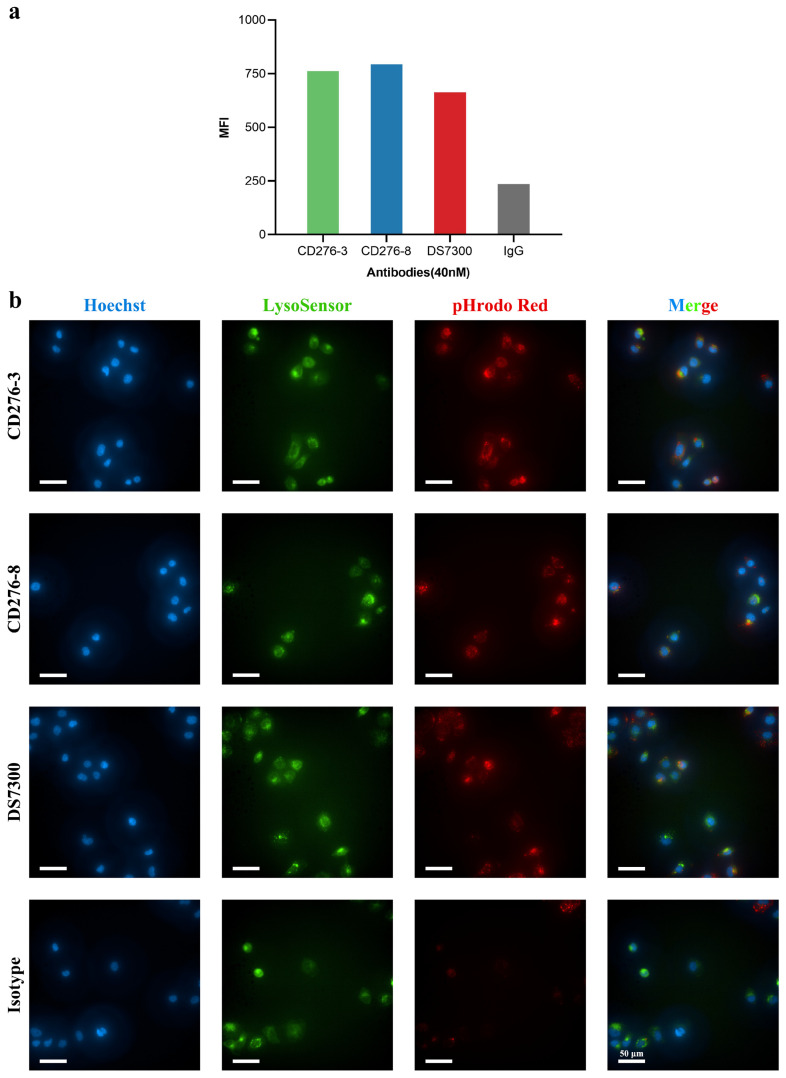
Internalization activity of CD276-3, CD276-8 and DS7300. Internalization assessment of CD276-3, CD276-8 and DS7300 (**a**) in U251 cells by FACS, (**b**) in OVCAR3 by HCA.

Considering that an antibody for ADC construction requires high internalization activity to deliver the payload into tumor cells, the internalization activity of candidates was assessed in vitro. Candidate antibodies and DS7300 were labeled with pHrodo Green and incubated with U251 cells for 24 h (Figure 2a). The labeled antibodies fluoresced green when the pH decreased and were detected using FACS, indicating that the antibodies were internalized into cells. Although CD276-8 showed moderate affinity compared to DS7300, it demonstrated better internalization activity than DS7300. Epitope differences may contribute to the internalization of CD276-8. In addition, the internalization activity of candidates was visually assessed in OVCAR3 using HCA (Figure 2b). After 4 h of incubation, red staining was observed intracellularly, indicating colocalization of antibodies with the green-stained lysosomes, which suggests that mAbs may be delivered intracellularly through lysosomes after receptor-mediated internalization.

### 2.3. In Vitro Cytotoxicity of CD276-3 ADC, CD276-8 ADC and DS7300 ADC (DAR8)

**Figure 3:**
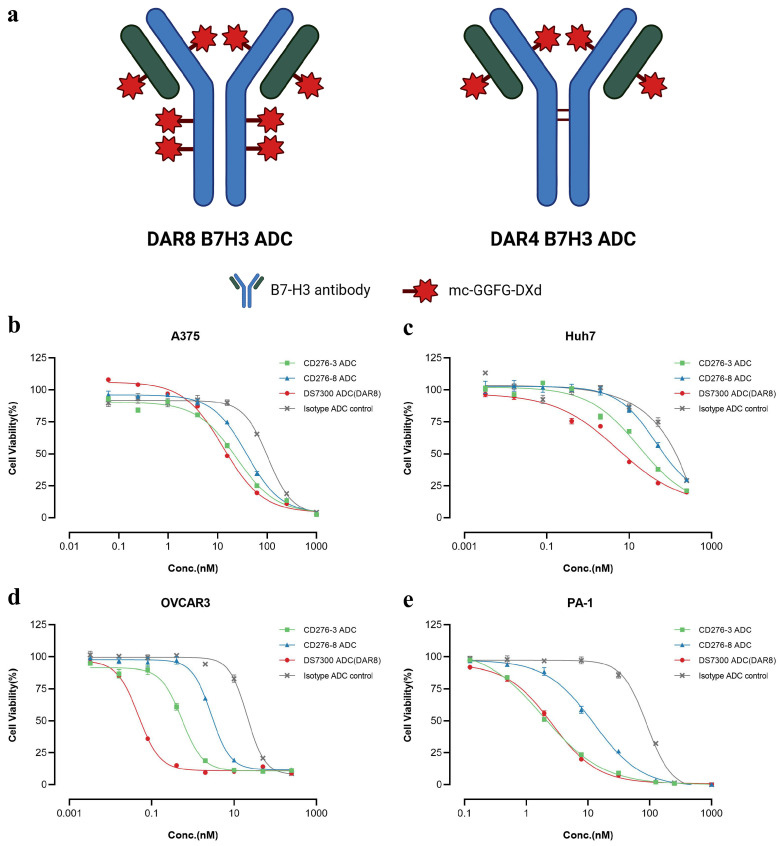
Structure of DAR8 and DAR4 ADCs and in vitro cytotoxicity of CD276-3 ADC, CD276-8 ADC and DS7300 ADC (DAR8). Created in BioRender. Ziyu, Z. (2026), https://BioRender.com/8ivqom2. (**a**) Chemical structure of DAR8 and DAR4 ADCs. The in vitro cytotoxicity was determined in positive cell lines, including A375 (**b**), Huh7 (**c**), OVCAR3 (**d**), and PA-1 (**e**). Each value represents the mean (M) and standard error of the mean (SEM) (n = 3).

After characterization, DAR8 ADCs, including CD276-3 ADC, CD276-8 ADC, DS7300 ADC and Isotype ADC, were generated by conjugating mc-GGFG-DXd and native cysteine residues of antibodies, while DS7300 ADC (DAR4) was generated by conjugating mc-GGFG-DXd to cysteine residues between the light and heavy chains of the antibody (Figure 3a). We used a site-specific conjugation approach with Zn^2+^ involved to generate the DAR4 DS7300 ADC. This approach enabled precise control over DAR and improved ADC homogeneity, thereby reducing systemic toxicity caused by inconsistent DAR values in subsequent studies [17,18].The purity of each ADC and antibody was detected by size exclusion chromatography (SEC-HPLC), and no clear differences between the antibodies and ADCs were observed (Δpurity ≤ 3%), as shown in Figure S3. The DARs of each ADC were detected by RP-HPLC. The DARs of DAR8 ADCs ranged from 7.26 to 7.56, and the DAR of DS7300 ADC (DAR4) was 4.42, meeting the expected DAR values (Figure S3) as designated.

The in vitro cytotoxicity of DAR8 ADCs was evaluated using several cell lines, and the IC_50_ values were concluded in Table 2. The IC_50_ of ADCs varied widely and showed no correlation with cell surface B7-H3 expression, as shown in Figure 1a. In B7-H3-positive expression cell lines, such as A375, Huh7, OVCAR3 and PA-1, all three ADCs showed specific tumor cell-killing (Figure 3b–e). In cells without B7-H3 expression, like Raji, no clear differences in IC_50_ were observed for all the ADCs (Table 2 and Table S1), indicating CD276-3 ADC and CD276-8 ADC exerted no B7-H3-specific cytotoxicity in negative cells. These results demonstrated that CD276-3 ADC and CD276-8 ADC showed B7-H3-specific cytotoxicity in vitro, with CD276-3 ADC showing better activity. However, some types of cancer cells were not sensitive to B7-H3 ADCs even though the cells highly expressed B7-H3, as detected by FACS, such as U251, which is the richest in B7-H3 expression, showing an IC_50_ over 200 nM or similar to isotype ADC (Table 2 and Table S1). The mechanisms causing the large differences in sensitivity could be related to cell-type-specific variations in target-mediated internalization, mitotic index, driver mutations, DNA repair capacity, apoptotic sensitivity, drug trafficking, drug metabolism, or efflux [13]. In addition, ADC requires a certain threshold of surface antigen expression to function effectively [19]. In this research, we only analyzed the relative B7-H3 expression level of the cell line. The absolute level of B7-H3 expression (antigen density) remained unclear. Therefore, cell lines exhibiting high relative expression levels but low antigen density may be selected for in vitro studies. These findings suggested that in vitro sensitivity screening and antigen density measurement could serve as a valuable approach for identifying cancer types sensitive to DXd-containing ADCs, aiding personalized therapy and helping ensure an optimal therapeutic window for DXd-containing ADCs [13].

### 2.4. Pharmacokinetics of the ADCs in Mice

**Figure 4:**
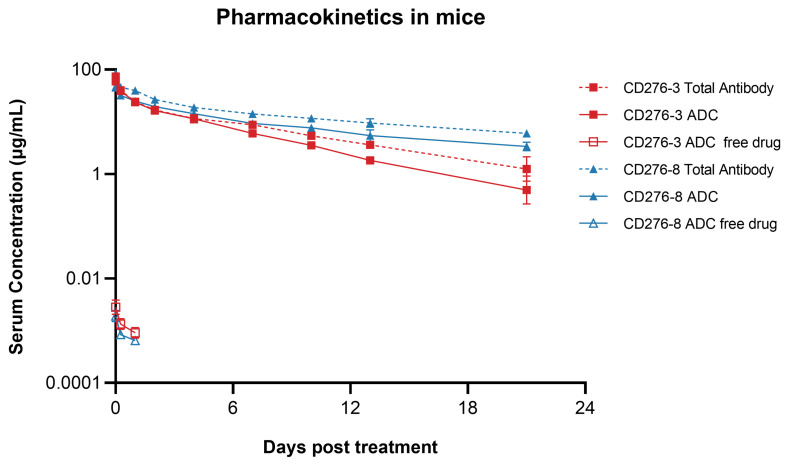
Pharmacokinetics of CD276-3 ADC and CD276-8 ADC in mice. CD276-3 ADC or CD276-8 ADC was administered once intravenously at 5 mg/kg to mice. Serum concentrations of ADC, total antibody and DXd were determined. Each value represents the mean and SEM (N = 5 mice per group).

CD276-3 ADC or CD276-8 ADC was administered once intravenously at 5 mg/kg to female NU/NU nude mice. Then, serum concentrations of ADC, total antibody and DXd were determined up to 21 days post-injection. The terminal elimination half-times (t_1/2_) of CD276-3 ADC and CD276-8 ADC were 4.20 and 7.30 days respectively, while the t_1/2_ of CD276-3 total antibody and CD276-8 total antibody were 5.91 and 9.94 days (Figure 4; Table 3). CD276-8 ADC showed a longer half-time (Figure 4; Table 3) and had the potential for long-lasting and potent tumor growth inhibition in vivo. Additionally, low serum concentrations of DXd were detected (Figure 4), indicating low systemic exposure to DXd attributed to the stable linker of CD276-3 ADC and CD276-8 ADC in comparison to previous reports [20,21,22]. Furthermore, no increase in the DXd concentration was observed even though CD276-8 ADC had a longer half-time, indicating considerable safety in mice (Figure 4).

### 2.5. Antitumor Activities of the ADCs In Vivo

**Figure 5:**
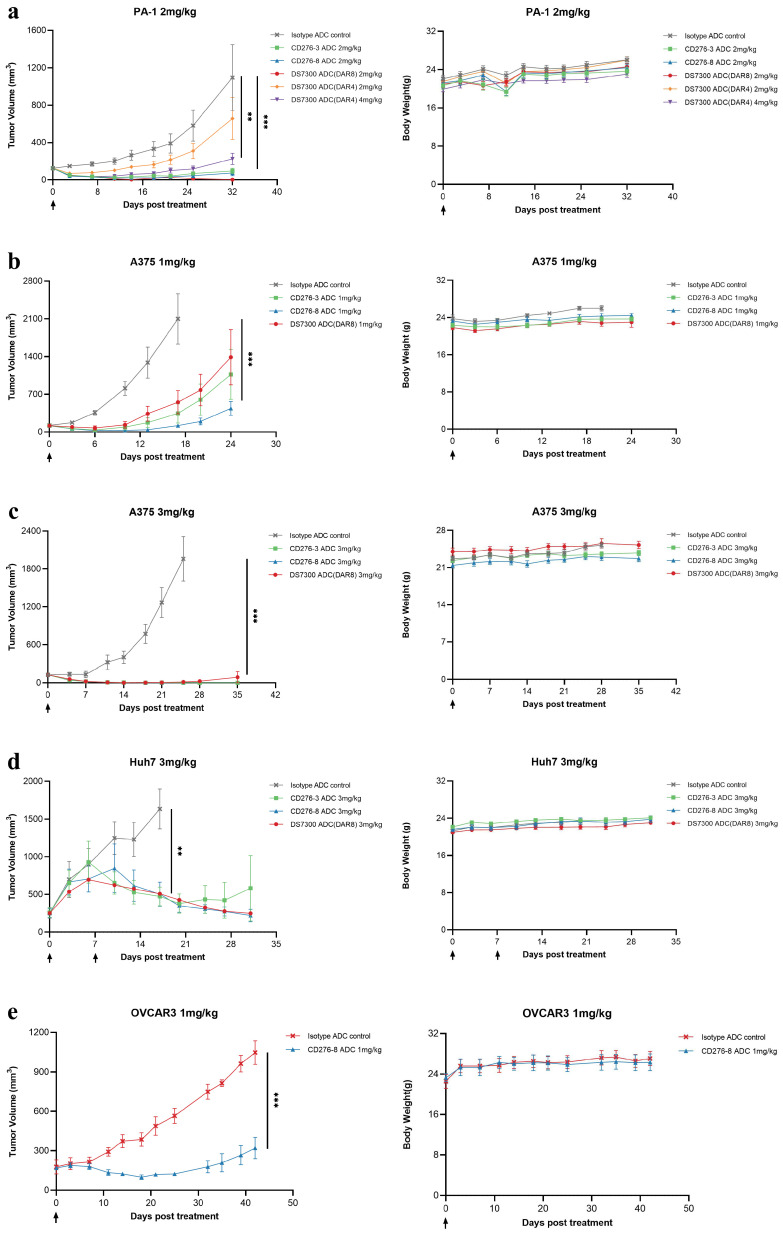
Antitumor activity of CD276-3 ADC, CD276-8 ADC and DS7300 ADC (DAR4 and DAR8) in CDX mouse models in vivo. (**a**) Mice with PA-1 cells were treated once with CD276-3 ADC, CD276-8 ADC, DS7300 ADC (DAR4), DS7300 ADC (DAR8) or isotype ADC control. (**b**,**c**) Mice with A375 cells were treated once with CD276-3 ADC, CD276-8 ADC, DS7300 ADC (DAR8) or isotype ADC control at 1 mg/kg (**b**) or 3 mg/kg (**c**). (**d**) Mice with Huh7 cells were treated with CD276-3 ADC, CD276-8 ADC, DS7300 ADC (DAR8) or isotype ADC control at 3 mg/kg on day 0 and day 7. (**e**) Mice with OVCAR3 were treated once with CD276-8 ADC or isotype ADC control. Body weight change of mice is shown for each study on the right. Each value represents the group mean and SEM. Statistical significance was calculated using unpaired one-way ANOVA with Tukey’s multiple comparison test and Student’s t-comparison test in GraphPad Prism (** *p* < 0.01, *** *p* < 0.001). The arrows below the horizontal axis indicate administration at that time point.

Given the varying sensitivity of different cell lines to B7-H3 ADCs, we selected cell lines sensitive to B7-H3 ADCs in vitro, such as PA-1, A375, Huh7 and OVCAR3, to construct CDX models in female NU/NU nude mice. Antitumor activities of CD276-3 ADC, CD276-8 ADC DS7300 ADC (DAR4) and DS7300 ADC (DAR8) were evaluated using CDX mouse models in vivo. CD276-8 ADC demonstrated potent antitumor activities (Figure 5a–e). In the PA-1 model (N = 6), 2 mg/kg candidates were compared with DS7300 ADC (DAR8), and DS7300 ADC (DAR4) was used as a reference representing the clinical format of ifinatamab deruxtecan. CD276-8 ADC induced more potent inhibition than CD276-3 ADC and DS7300 ADC (DAR4), with a tumor growth inhibition (TGI, %) of 93.1% compared to isotype ADC at 32 days, and 2 of 6 maintaining complete tumor inhibition until the end of the study (Figure 5a). However, DS7300 ADC (DAR8) demonstrated slight dominance compared to CD276-8 ADC, reaching a TGI of 99.8%.

Furthermore, the antitumor activities of CD276-3 ADC, CD276-8 ADC and DS7300 ADC (DAR8) were determined at different doses in A375 and Huh7 CDX mouse models (N = 6). In the A375 model, CD276-8 ADC induced dose-dependent antitumor activity with TGIs of 94.3% (1 mg/kg) and 100% (3 mg/kg), while the TGIs of DS7300 ADC (DAR8) were 73.7% (1 mg/kg) and 99.3% (3 mg/kg) (Figure 5b,c). In the Huh7 model, CD276-8 ADC demonstrated durable tumor regression out to day 30, and DS7300 ADC (DAR8) showed tumor inhibition activity with comparable durability and potency (Figure 5d). Finally, CD276-8 ADC was chosen as the ultimate ADC molecule. And its antitumor activity was further determined in the OVCAR3 model, in which it also showed potent tumor inhibition with a TGI of 69.4% on day 42 (Figure 5e). The body weight of the mice treated with CD276-8 ADC in all models showed no obvious reduction at the doses and dosing frequencies used in the experiment (Figure 5a–e).

Taken together, these results suggest that CD276-8 ADC induced a potent and long-lasting antitumor activity comparable to DS7300 ADC (DAR8), while CD276-3 ADC appeared weaker in vivo, although it demonstrated potent cytotoxicity in vitro. Therefore, CD276-8 ADC was chosen as the ultimate ADC molecule for further development.

### 2.6. Developability Assessment of CD276-8 ADC

After comparisons between CD276-3 ADC and CD276-8 ADC in vivo and in vitro, the developability of CD276-8 ADC was assessed.

The parental antibody CD276-8 was characterized to evaluate its colloidal stability and nonspecific interaction compared to a standard sample, Tremelimumab [23]. CD276-8 showed a shorter retention time (RT) of 8.992 min in SMAC-HPLC and 8.355 min in CIC-HPLC, while the RT of Tremelimumab was 21.129 min in SMAC-HPLC and 13.088 min in CIC-HPLC, demonstrating a low risk of colloidal instability and low nonspecific interaction [23,24] (Figure S4a,b). Furthermore, AS and F/T tests of CD276-8 were analyzed by SEC-HPLC and WCX-HPLC. No clear purity change was observed by SEC-HPLC, demonstrating low aggregation of CD276-8 (Figure S4c). A low ratio of acidic peak of CD276-8 was observed by WCX-HPLC and around 10% of acidic variant was detected after 14 days of incubation at 40 °C (Figure S4d), which demonstrated that CD276-8 has a low risk of charge heterogeneity [25]. The melting point (T_m_) of CD276-8 detected by Differential Scanning Fluorimetry (DSF) was 68.8 °C (Figure S4e), showing acceptable thermal stability [26]. These data indicated that CD276-8 is a stable antibody with good developability.

**Figure 6:**
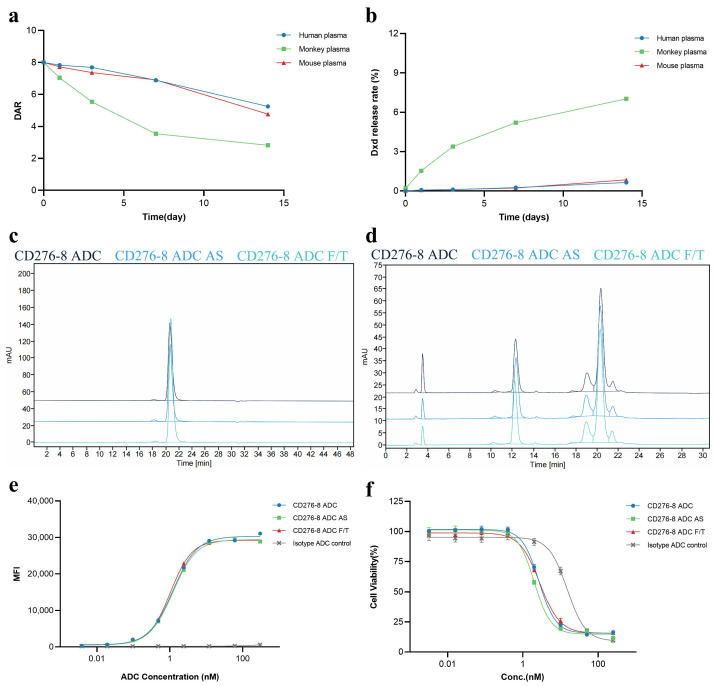
Developability of CD276-8 ADC. (**a**,**b**) DAR change and DXd release rate of CD276-8 ADC in various types of plasma after incubation up to 14 days, as detected by LC-MS. (**c**) Purity of CD276-8 ADC sample from AS or F/T study was assessed by SEC-HPLC. (**d**) DAR of CD276-8 ADC sample from AS or F/T study was assessed by RP-HPLC. (**e**) Binding affinity of CD276-8 ADC sample from AS or F/T study was determined using OVCAR3 cells by FACS. (**f**) Cytotoxicity of CD276-8 ADC sample from AS or F/T study was determined using OVCAR3 cells by SpectraMax iD5.

Furthermore, the T_m_ of CD276-8 ADC was 69.8 °C and 56.6 °C, as detected by DSF (Figure S4f). Considering that the interchain disulfide bonds of CD276-8 were reduced for linker-payload conjugation, the thermal stability change was acceptable. Plasma stability was evaluated in vitro in terms of DAR change and DXd release rate in human, monkey and mouse plasma. The DAR change rates of 0.2 mg/mL CD276-8 ADC after 14 days of incubation in human, monkey and mouse plasma were 34.4%, 64.7% and 40.4% (Figure 6a). And the DXd release rates of CD276-8 ADC after 14-day incubation in human, monkey and mouse plasma were 0.65%, 7.02% and 0.85% (Figure 6b). The stability profiles of CD276-8 ADC were similar to the previously reported ADC using mc-GGFG-DXd [27,28]. The substantial DAR change and DXd release rate in monkey plasma reminded us to pay closer attention to potential systemic toxicity in monkeys in our further studies. Regarding the stability of CD276-8 ADC, structural stability and biological activity were evaluated. No significant alterations were observed in purity and DAR (Figure 6c,d), indicating CD276-8 ADC was stable after 14 days of incubation at 25 °C or 5 cycles of freezing and thawing. The EC_50_ of cell binding affinity and the IC_50_ of in vitro cytotoxicity of CD276-8 ADC showed no significant decrease under stability testing, further confirming its stability in vitro. Taking all data together, CD276-8 ADC shows acceptable developability and is worth further development.

## 3. Discussion

Here, we report the development of CD276-8 ADC, a potent DXd-based ADC with moderate affinity targeting B7-H3. CD276-8 ADC demonstrated potent and long-lasting antitumor activities in B7-H3-positive CDX models although showing less potent cytotoxicity in vitro compared with DS7300 ADC and CD276-3 ADC.

B7-H3, the target of CD276-8 ADC, is a membrane protein with low level of expression on normal human tissues but with higher levels of expression on various solid tumors including NSCLC, pancreatic cancer, hepatocellular carcinoma, breast carcinoma, prostate adenocarcinoma, and melanoma [2,4,5,6]. The expression pattern with unmet clinical need indicates that B7-H3 is a promising target for ADC therapy with potential potent antitumor activities and minimal non-specific systemic toxicity.

Compared with CD276-3 ADC and benchmark molecule DS7300 ADC (DAR8), CD276-8 ADC showed a moderate binding affinity against B7-H3 and a weaker cytotoxicity in various B7-H3-positive cell lines in vitro. As expected, CD276-8 ADC showed no B7-H3-specific cytotoxicity in B7-H3-negative cells like Raji. Although all of the tested cells were positive for the free payload DXd, CD276-8 ADC exhibited target-specific cytotoxicity only in some types of tumor cells but no target-specific cytotoxicity in others. The mechanisms causing significant differences in sensitivity could be related to cell-type-specific variations in target-mediated internalization, mitotic index, driver mutations, DNA repair capacity, apoptotic sensitivity, drug trafficking, drug metabolism, or efflux [13]. Additionally, the lack of absolute B7-H3 expression levels in cell lines may result in the involvement of insensitive cell lines in the study [19]. These results suggested the value of in vitro sensitivity screening and antigen density measurement as a tool for identifying cancer types sensitive to DXd-containing ADCs and ensuring an optimal therapeutic window for DXd-containing ADCs [13].

A pivotal finding of our study was the inconsistency between in vitro and in vivo activities, highlighting a non-linear relationship between affinity and overall ADC efficacy, including in vitro cytotoxicity, pharmacokinetic profiles and in vivo antitumor activity. Although CD276-8 ADC exhibited moderate binding affinity and correspondingly weaker cytotoxicity in monolayer cell cultures compared to CD276-3 ADC and the high-affinity benchmark DS7300 ADC (DAR8), it achieved superior or comparable tumor regression in vivo. Preliminary pharmacokinetic assessment in mice suggested a relatively long half-life and limited systemic exposure to free DXd, which may contribute to sustained antitumor activity while potentially reducing off-target toxicity risk. However, we acknowledge that more comprehensive pharmacokinetic studies are needed to fully characterize the disposition of CD276-8 ADC.

The observed discrepancy between in vitro and in vivo performance may be attributed to multiple factors. First, previous studies have reported that excessively high affinity can impede antibody penetration into solid tumors due to the “binding site barrier” effect, while moderate affinity has been proposed to facilitate more uniform intratumoral distribution and enhance the bystander effect [29,30,31]. CD276-8 ADC with moderate-affinity may exhibit better distribution in tumor tissues. Second, CD276-8 bound B7-H3 on a different epitope compared with CD276-3 and DS7300, showing better internalization and affecting lysosomal delivery efficiency and subsequent payload release. Third, lysosomal processing may be different in vivo, where tumor cells are exposed to acidic conditions. This difference may promote the secretion of active cathepsin B and thus promote the cleaving of GGFG linker and release of DXd in tumor cells in vivo [32]. Fourth, based on our pharmacokinetic assessment, the longer half-life of CD276-8 ADC in mice may result in a greater amount of intact ADC reaching the tumor compared to CD276-3 ADC. Finally, patterns of drug distribution and target engagement can be influenced by interstitial pressure and heterogeneous vascular perfusion in solid tumors, which cannot be recapitulated in vitro [33,34]. These tumor microenvironment-specific factors, combined with the moderate-affinity design, may collectively explain why CD276-8 ADC demonstrates potent in vivo efficacy despite moderate activity in standard cytotoxicity assays. Further studies are warranted to delineate the relative contributions of these mechanisms.

Based on the combination of in vivo efficacy, preliminary pharmacokinetic observations, and acceptable developability, we selected CD276-8 ADC as our lead candidate for further development. Although the mechanistic hypotheses discussed above have been supported by previous literature, we recognize that direct experimental validation is still required, such as tumor-section imaging, quantitative biodistribution analysis, and more comprehensive pharmacokinetic studies, to definitively establish the underlying mechanisms. These investigations represent important directions for our future studies.

In summary, we developed CD276-8 ADC, a novel B7-H3-targeting ADC conjugated with the potent TOP1 inhibitor DXd. CD276-8 ADC demonstrated potent antitumor activity in preclinical CDX models, accompanied by acceptable pharmacokinetic profiles and acceptable developability. The moderate-affinity design of CD276-8 ADC may contribute to its in vivo performance, potentially by facilitating tumor penetration and reducing target-mediated clearance, as suggested in previous studies. But the precise mechanisms underlying its efficacy remain to be fully elucidated. Our findings offer CD276-8 ADC as a promising therapeutic candidate for patients with B7-H3-expressing solid tumors and warrant further investigation into its mechanism of action and safety profile.

## 4. Materials and Methods

### 4.1. Antibodies and ADCs

The scFv sequences of parental anti-B7-H3 antibodies (Ab) CD276-3 and CD276-8 were screened from a human-derived ScFv phage display antibody library using phage display technology and were used to construct full-length IgG1 mAb molecules. The CDR sequences of DS7300a parental antibody [35] (described as DS7300 in this study) were from US11633493B2 [36], and its structure is the same as that of parental anti-B7-H3 antibodies. DS7300 was used as a positive control in the study.

To produce CD276-3 ADC, CD276-8 ADC, DS7300 ADC and isotype ADC control with an average DAR of 8, antibodies with native cysteine residues were conjugated to the linker-payload mc-GGFG-DXd composed of DXd and a maleimide-GGFG peptide by a standard method [11,37]. Antibodies were reduced by a 12-fold concentration of TCEP at 37 °C for 3 h and then conjugated with a 13-fold concentration of linker-payload at room temperature for 1 h. The reaction was terminated by excess L-Cysteine.

In addition, DS7300 ADC with an average DAR of 4 was produced in the presence of transition metal ion Zn^2+^ to improve the homogeneity of ADC [18]. DS7300 was reduced by a 6-fold concentration of TCEP at 4 °C overnight with 2-fold ZnCl_2_ involved. Then it was conjugated with a 12-fold concentration of linker-payload at 4 °C for 2 h. The reaction was terminated by excess L-Cysteine, and EDTA buffer was added to capture Zn^2+^. Finally, dehydroascorbic acid (DhAA) was added to oxidize the DS7300 antibody.

The purities of antibodies and ADCs were characterized by SEC-HPLC column TSKgel G3000SWxl (300 Å, 7.8 × 300 mm, Tosoh Bioscience, Tokyo, Japan) using phosphate-buffered saline (PBS, pH 7.4) as the mobile phase. The DAR of ADCs was detected by RP-HPLC column PLRP-S (4.6 × 250 mm, 8 μm, 1000 Å, Agilent, Santa Clara, CA, USA) using ultrapure water and acetonitrile with 0.1% trifluoroacetic acid (TFA) as mobile phases A and B, with a linear gradient of 30–41% phase B from 0 min to 20 min, 41–45% phase B from 20 min to 25 min, and 30% phase B from 25 min to 35 min [38]. All HPLC in the study was conducted on 1260 Infinity II (Agilent). The DAR of each ADC was calculated using the formula

DAR = 2 × (Σ_LC Weighted peak area_ + Σ_HC Weighted peak area_)/100

### 4.2. Cell Lines

The human glioma cell line U251 (RRID: CVCL_0021), the human gastric carcinoma cell NCI-N87 (RRID: CVCL_1603), the human ovarian carcinoma cell OVCAR3 (RRID: CVCL_0465), and the human pancreatic adenocarcinoma cell BxPC-3 (RRID: CVCL_0186) were purchased from ATCC. The human epidermoid carcinoma cell A431 (RRID: CVCL_0037) and the human lung adenocarcinoma cell HCC827 (RRID: CVCL_2063) were purchased from Hysigen Bioscience (Suzhou, China). The human ovarian teratoma cell PA-1 (RRID: CVCL_0479) was purchased from Procell Life Science&Technology Co., Ltd. (Wuhan, China). The human melanoma cell line A375 (RRID: CVCL_0132), the human breast cancer cells MCF-7 (RRID: CVCL_0031), MDA-MB-231 (RRID: CVCL_0062), the human liver hepatocellular carcinoma cells HepG2 (RRID: CVCL_0027), Huh7 (RRID: CVCL_0336), and the human Burkitt’s lymphoma cells Raji (RRID: CVCL_0511) and Daudi (RRID: CVCL_0008) were kept in Jecho Institute Co., Ltd. (Shanghai, China). All of the cells were cultured with appropriate media.

### 4.3. B7-H3 Expression Analysis by FACS

Each cell line was treated with DS7300 or human IgG1 isotype control, and was stained with PE-conjugated F(ab’)2-goat anti-human IgG Fc (Invitrogen, Shanghai, China). Each sample was analyzed by Attune NxT (ThermoFisher, Shanghai, China), and the expression of cell surface B7-H3 was evaluated in the form of MFI folds in comparison with the isotype control.

### 4.4. Bio-Layer Interferometry Assay

Recombinant human B7-H3 (rhB7-H3) protein (4Ig B7-H3, His tag) (Cat: 11188-H08H, Sino Biological, Inc., Beijing, China), composed of the full-length extracellular domain of human B7-H3 and a poly-histidine tag at the C-terminus, was used in the BLI assay to represent the native human B7-H3 protein. The binding affinities of CD276-3, CD276-8 and DS7300 with rhB7-H3 protein were analyzed by BLI with Gator PRIME (Gator Bio, Shanghai, China). In total, 6 μg/mL rhB7-H3 protein with a His tag was immobilized to Ni NTA probes (Gator Bio) and was associated with a series of gradient concentrations (from 25 μg/mL to 1.5625 μg/mL) of CD276-3, CD276-8 or DS7300 for 5 min to determine the association curves. Then, probes were placed in phosphate buffered saline with Tween 20 (1 × PBST) to determine the dissociation curves. The K_D_ of antibodies was calculated by the software. The protein and antibodies were dissolved in 1 × PBST.

Furthermore, epitope grouping of CD276-3, CD276-8 and DS7300 was processed. In total, 6 μg/mL rhB7-H3 protein was immobilized to a Ni NTA probe and was associated with 20 μg/mL CD276-3, CD276-8 or DS7300. After the first antibody association, the probe was associated with 20 μg/mL CD276-3, CD276-8 or DS7300 separately. The binding response of each mAb was determined as Ab1 and Ab2 to obtain Shift 1 (nm) and Shift 2 (nm), and the inhibition rates of Ab1 against Ab2 were calculated with 100% × (1-(Shift 2 of Ab1 with Ab2 as the first antibody)/(Shift 1 of Ab1)). The protein and antibodies were dissolved in 1 × PBST.

### 4.5. Cell Binding Affinity Analysis

The binding affinity against cell surface antigen of CD276-3, CD276-8 or DS7300 was evaluated by FACS. U251 or A375 cells were seeded into 96-well cell culture plates at a concentration of 1.5 × 10^5^ cells per well. CD276-3 CD276-8, DS7300 or human IgG isotype control was serially diluted and incubated with cells for 30 min at 4 °C. Then, the cells were washed and incubated with PE-conjugated F(ab’)2-goat anti-human IgG Fc for 30 min at 4 °C. After washing and resuspension, samples were analyzed by Attune NxT. The EC_50_ value was calculated in GraphPad Prism using a nonlinear, 4-parameter curve fit model to determine the binding affinity against cell surface B7-H3 of CD276-3, CD276-8 and DS7300.

### 4.6. Cell Internalization Evaluation

Internalization of CD276-3, CD276-8 or DS7300 was evaluated by FACS. U251 cells were seeded into 96-well cell culture plates at 1 × 10^4^ cells per well and incubated overnight at 37 °C in a humidified atmosphere containing 5% CO2. CD276-3, CD276-8, DS7300 or human IgG isotype control was mixed with pHrodo iFL Green (Invitrogen), and then incubated with cells at 37 °C for 24 h in a humidified atmosphere containing 5% CO_2_. The internalization condition was detected with standard FITC using Attune NxT.

Furthermore, antibodies conjugated with pHrodo Red (Invitrogen) were visualized in cell plasma by HCA. OVCAR3 cells (1 × 10^4^ cells/well) were dosed with 0.1 mg/mL antibodies conjugated with pHrodo Red at 37 °C for 4 h. Treated cells were washed with PBS to remove unbound antibodies. In addition to the signal of antibody-pHrodo, treated cells were visualized with Lysosensor Green (Invitrogen) and Hoechst Blue [39]. The stained cells were detected by Operetta CLS (Perkin Elmer Shanghai, China), and the co-positioning situation was analyzed using ImageJ 1.54.

### 4.7. In Vitro Cytotoxicity

The cells were seeded into 96-well cell culture plates at 500 to 2000 cells per well. After overnight incubation at 37 °C, each serially diluted substance (CD276-3 ADC, CD276-8 ADC, DS7300 ADC (DAR8) and isotype ADC control) was added, and then all the plates were incubated for 5 days. After incubation, cell viability was detected by SpectraMax iD5 (Molecular Devices, LLC, Shanghai, China) using CellTiter-Glo (CTG, Promega Corp., Beijing, China). The half-maximal inhibitory concentration (IC_50_) of each substance was calculated in GraphPad Prism using a nonlinear, 4-parameter curve fit model.

### 4.8. In Vivo Activity

All animal experiments performed in this study were reviewed and approved by the Institutional Animal Care and Use Committees of the Laboratory Animal Center, Shanghai Jiao Tong University.

#### 4.8.1. Pharmacokinetic Study in Mice

CD276-3 ADC or CD276-8 ADC was injected once intravenously at 5 mg/kg into female NU/NU nude mice. Serum concentrations of ADC, total antibody (drug conjugated and unconjugated antibody), and DXd were measured up to 21 days post-treatment. Concentrations of total antibody and ADC were detected by ELISA [40], and DXd were detected by LC-MS using Xevo TQ-XS (Waters Corp., Shanghai, China) [28]. The lower limit of quantification (LLOQ) for ADC and total antibody was 0.137 ug/mL, and the LLOQ for DXd was 0.1 ng/mL. The pharmacokinetic parameters were calculated by noncompartmental analysis with PKSolver 2.0 [41].

#### 4.8.2. Tumor Inhibition in Cancer Cell Line-Derived Xenograft Models

A375, Huh7 and OVCAR3 models were established by injecting 5 × 10^6^ cells suspended in RPMI 1640, while the PA-1 model was established by injecting 9 × 10^6^ cells suspended in RPMI 1640, subcutaneously into female NU/NU nude mice (Vital River Laboratory Animal Technology Co., Ltd., Jiaxing, China).

When the tumor volume reached approximately 100–300 mm^3^, the mice were randomly distributed into study groups. Tumor-bearing mice were treated with CD276-3 ADC, CD276-8 ADC, DS7300 ADC (DAR4 or DAR8), or Isotype ADC control intravenously on day 0. Mice in the Huh7 model were treated with a second dose on day 7. The dose range was preliminarily determined based on prior studies using the same linker payload and DAR values [22]. And we made appropriate adjustments within the predetermined dose range according to the specific sensitivity of each cell line. The experiments were conducted as shown in Table S2. The tumor volume and weight were measured twice a week, and the tumor volume was defined as 0.5 × tumor length × tumor width^2^. The antitumor activity was evaluated when the tumor volume of the Isotype ADC control group reached 2000 mm^3^ (1000 mm^3^ in the case of PA-1 and OVCAR3 models). In addition, the TGI was calculated using the formula

TGI = 100 × (1 − (V_T_)/(Vc))

V_T_: average tumor volume of treatment group
Vc: average tumor volume of control group

### 4.9. Developability Assessment of the ADC

#### 4.9.1. Characterization of CD276-8

The purity and colloidal stability of CD276-8 were characterized by a standup monolayer adsorption chromatography (SMAC-HPLC) column Zenix SEC-300 (300 Å, 4.6 × 300 mm, Sepax) using 0.1 M phosphate buffer and 0.1 M Na_2_SO_4_ solution (pH 6.7) as the mobile phase [23]. The nonspecific interaction of CD276-8 was characterized by cross interaction chromatography (CIC-HPLC) prepared by coupling 30 mg of human serum polyclonal antibodies (I4506; Sigma) to a 1-mL HiTrap column (GE Healthcare, Shanghai, China) and quenching with ethanolamine [24]. PBS (pH 7.0) was used as the mobile phase. In addition, the thermal stability of CD276-8 was characterized by DSF in the form of T_m_.

#### 4.9.2. Stability of CD276-8 and CD276-8 ADC in Forced Degradation

Stability of CD276-8 was evaluated by accelerated stability (AS) test and freeze-thawing (F/T) test. In the AS test, CD276-8 in pH 5.5 PBS was placed in a 40 °C water bath for 14 days, while in the F/T test, CD276-8 was frozen at −80 °C and thawed at 25 °C for 5 cycles. The aggregation of each sample was characterized by SEC-HPLC. And the charge variants were detected by weak cation exchange chromatography (WCX-HPLC) column ProPac WCX-10 (9 × 250 mm, 10 μm, ThermoFisher) using 20 mM MES buffer (pH 6.7, phase A) and 20 mM MES buffer (pH 6.7, 0.5 M NaCl, phase B) as the mobile phase with a linear gradient of 5–50% phase B from 3 min to 30 min and 80% phase B from 30 min to 40 min.

In the AS test of CD276-8 ADC, CD276-8 ADC in histidine buffer (pH 5.5) was placed in a 25 °C water bath for 2 weeks. The F/T test of CD276-8 ADC was conducted using the same method as for CD276-8. For each sample, aggregation was detected by SEC-HPLC using a method mentioned in Section 4.1, and the DAR of CD276-8 ADC was detected by RP-HPLC using a method mentioned in Section 4.1. In the case of ADC biological activities, the cell binding affinity and in vitro cytotoxicity changes between samples were measured by FACS and SpectraMax iD5 using OVCAR3 cells.

#### 4.9.3. In Vitro Plasma Stability of CD276-8 ADC

CD276-8 ADC was diluted into human, monkey and mouse plasma to yield a final solution with 0.2 mg/mL CD276-8 ADC, and the sample was incubated at 37 °C. Plasma concentration of DXd and DAR of CD276-8 ADC were measured for up to 14 days by LC-MS using Xevo G2-XS QTof and Xevo TQ-XS (Waters Corp.) [28]. The BLQ for DXd was 0.1 ng/mL.

## Figures and Tables

**Table 1:** Binding affinity against rhB7-H3 (4Ig) of CD276-3, CD276-8 and DS7300 as determined by BLI.

Antibody	K_off_ (1/s)	K_on_ (1/Ms)	K_D_ (nM)
CD276-3	1.74 × 10^−3^	1.41 × 10^6^	1.24
CD276-8	7.02 × 10^−4^	3.31 × 10^5^	2.12
DS7300	2.35 × 10^−4^	1.02 × 10^6^	0.231

**Table 2:** In vitro cytotoxicity (IC_50_) of ADCs in various cell lines as calculated by GraphPad Prism 8.

		IC_50_ (nM)			
Cell Line	Cancer Type	CD276-3 ADC	CD276-8 ADC	DS7300 ADC (DAR8)	Isotype ADC
Raji	lymphoma	51.99	52.14	55.13	61.31
U251	glioma	>200	>200	>200	>200
A375	melanoma	23.91	38.67	12.97	104.1
A431	skin cancer	176.7	>200	196.2	>200
MDA-MB-231	breast cancer	>200	>200	>200	>200
NCI-N87	stomach cancer	>200	>200	>200	>200
HepG2	liver cancer	>200	>200	>200	>200
Huh7	liver cancer	17.39	43.69	5.265	>200
HCC827	lung cancer	21.73	15.04	7.746	38.72
OVCAR3	ovarian cancer	0.5388	2.802	0.04758	20.73
PA-1	ovarian teratoma	1.897	12.43	2.613	89.76

**Table 3:** Pharmacokinetic parameters of CD276-3 ADC and CD276-8 ADC in mice.

Pharmacokinetic Parameter *	CD276-3 ADC	CD276-3 Total Ab	CD276-8 ADC	CD276-8 Total Ab
AUC_inf_ (μg·day/mL)	152.74 ± 14.73	196.13 ± 22.24	245.26 ± 30.14	414.11 ± 43.84
AUC_21d_ (μg·day/mL)	147.64 ± 13.99	179.54 ± 19.77	206.24 ± 19.95	317.24 ± 18.25
CL (mL/day/kg)	33.88 ± 3.01	26.57 ± 2.40	21.77 ± 2.85	12.65 ± 1.37
t_1/2_ (day)	4.20 ± 0.50	5.91 ± 0.84	7.30 ± 1.27	9.94 ± 1.55
V_ss_ (mL/kg)	171.74 ± 23.16	196.69 ± 25.41	197.66 ± 8.41	158.84 ± 7.45
MRT_inf_ (day)	5.07 ± 0.42	7.47 ± 0.77	9.85 ± 1.50	13.38 ± 1.91

* The parameters were calculated by noncompartmental analysis with PKSolver, and each value was presented in the form of Mean ± SEM (N = 5 mice per group).

## Data Availability

The original contributions presented in this study are included in the article and Supplementary Material. Further inquiries can be directed to the corresponding author(s).

## Supplementary Materials

The following supporting information can be downloaded at https://www.mdpi.com/article/10.3390/ph19040596/s1: Figure S1: Binding affinity curves of CD276-3, CD276-8, and DS7300 against human B7-H3; Figure S2: The epitope grouping of CD276-3, CD276-8 and DS7300 determined by BLI; Figure S3: Characterization of CD276-3 ADC, CD276-8 ADC, DS7300 ADC (DAR8), DS7300 ADC (DAR4) and isotype ADC; Figure S4: The developability of CD276-8 and thermal stability of CD276-8 and CD276-8 ADC. Table S1: Summary of in vitro cytotoxicity (IC50) of B7-H3 ADCs in various cell lines. Table S2: Summary of in vivo antitumor experiment conditions in different CDX mouse models.

## Abbreviations

The following abbreviations are used in this manuscript:

ADC	Antibody–Drug Conjugate
BLI	Bio-Layer Interferometry
HCA	High-Content Analysis
CDX	Cell Line-Derived Xenograft
TOP1	Topoisomerase I
IgC	Immunoglobulin Constant-like Domain
IgV	Immunoglobulin Variable-like Domain
NSCLC	Non-Small Cell Lung Cancer
IHC	Immunohistochemistry
TME	Tumor Microenvironment
ADCC	Antibody-Dependent Cellular Cytotoxicity
mAb	Monoclonal Antibody
TCE	T-Cell Engager
CAR-T	Chimeric Antigen Receptor T Cell
mc-GGFG-DXd	Deruxtecan
MFI	Median Fluorescence Intensity
FACS	Fluorescence Activated Cell Sorting
HPA	Human Protein Atlas
PE	R-Phycoerythrin
rhB7-H3	Recombinant Human B7-H3
EC_50_	Concentration for 50% of Maximal Effect
DAR	Drug-to-Antibody Ratio
SEC-HPLC	Size Exclusion Chromatography
RP-HPLC	Reversed-Phase High-Performance Liquid Chromatography
SEM	Standard Error of Mean
IC_50_	Half-Maximal Inhibitory Concentration
TGI	Tumor Growth Inhibition
SMAC-HPLC	Standup Monolayer Adsorption Chromatography
CIC-HPLC	Cross Interaction Chromatography
AS	Accelerated Stability
F/T	Freeze-Thawing
T_m_	Melting Point
DSF	Differential Scanning Fluorimetry
CDR	Complementarity Determining Region
CTG	CellTiter-Glo
BLQ	Below Lower Limit of Quantification
PBS	Phosphate Buffered Saline
WCX-HPLC	Weak Cation Exchange Chromatography
MES	2-Morpholinoethanesulphonic acid
TFA	Trifluoroacetic Acid
RT	Retention Time
