# Supplementary material for: A Moderate-Affinity Antibody–Drug Conjugate Targeting B7-H3 Exerts Potent Antitumor Efficacy
Source: Pharmaceuticals (Basel). 2026 Apr 8;19(4):596. doi: 10.3390/ph19040596 (PMC13118972; doi:10.3390/ph19040596)
Supplement: Supplementary file 1 [file pharmaceuticals-19-00596-s001.zip › pharmaceuticals-4163747-supplementary.pdf]

Supplemental Material: A moderate-affinity antibody–drug conjugate targeting B7-H3 exerts potent antitumor efficacy

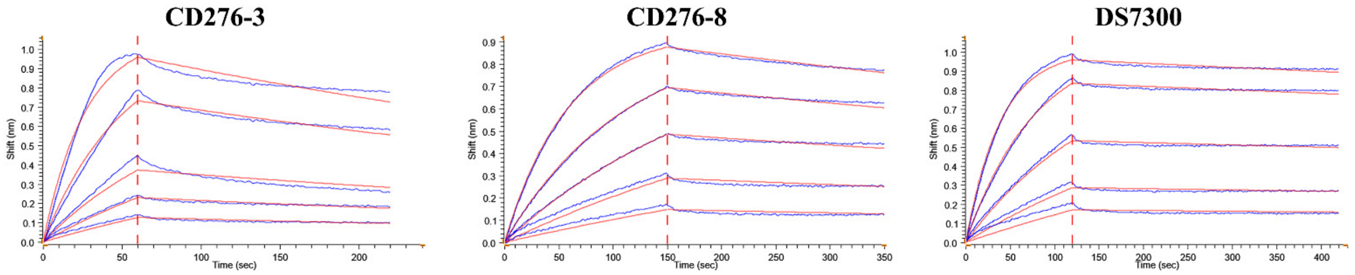

Figure S1. Binding affinity curves of CD276-3, CD276-8, DS7300 against human B7-H3. The binding affinity of each candidate was determined by Bio-Layer Interferometry (BLI). The blue curves represent sensorgrams of different concentrations of the analytes used in BLI analysis. The red curves represent the fitted curves for K_D calculation of analytes.

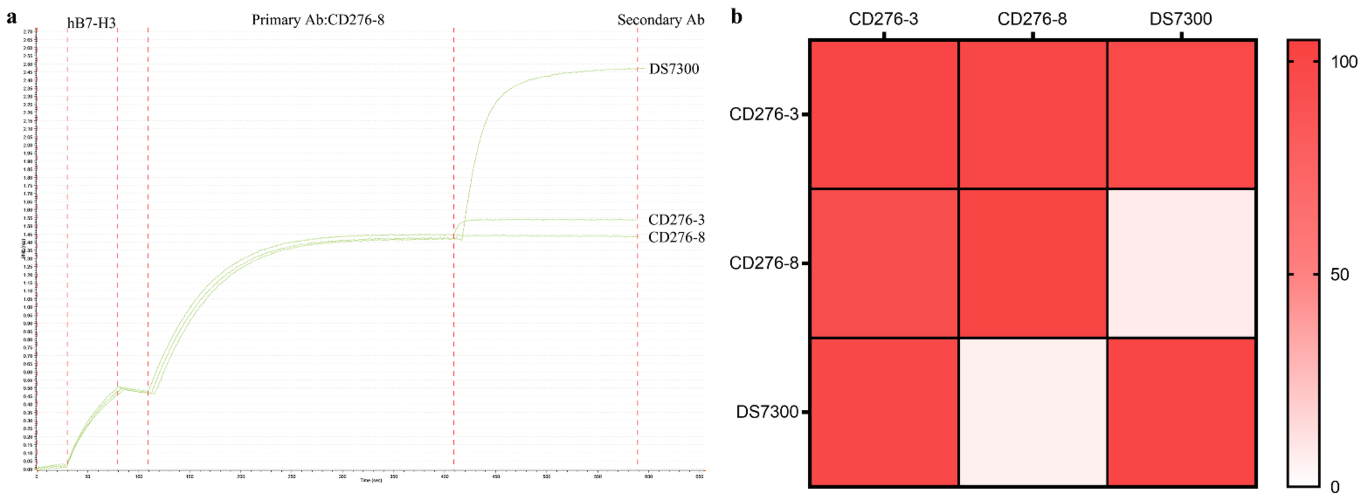

Figure S2. The epitope grouping of CD276-3, CD276-8 and DS7300 determined by BLI. **(a)** Sensorgrams of Different secondary antibodies including CD276-3, CD276-8 and DS7300, with CD276-8 as primary antibody. **(b)** Inhibition rate heatmap of CD276-3, CD276-8 and DS7300.

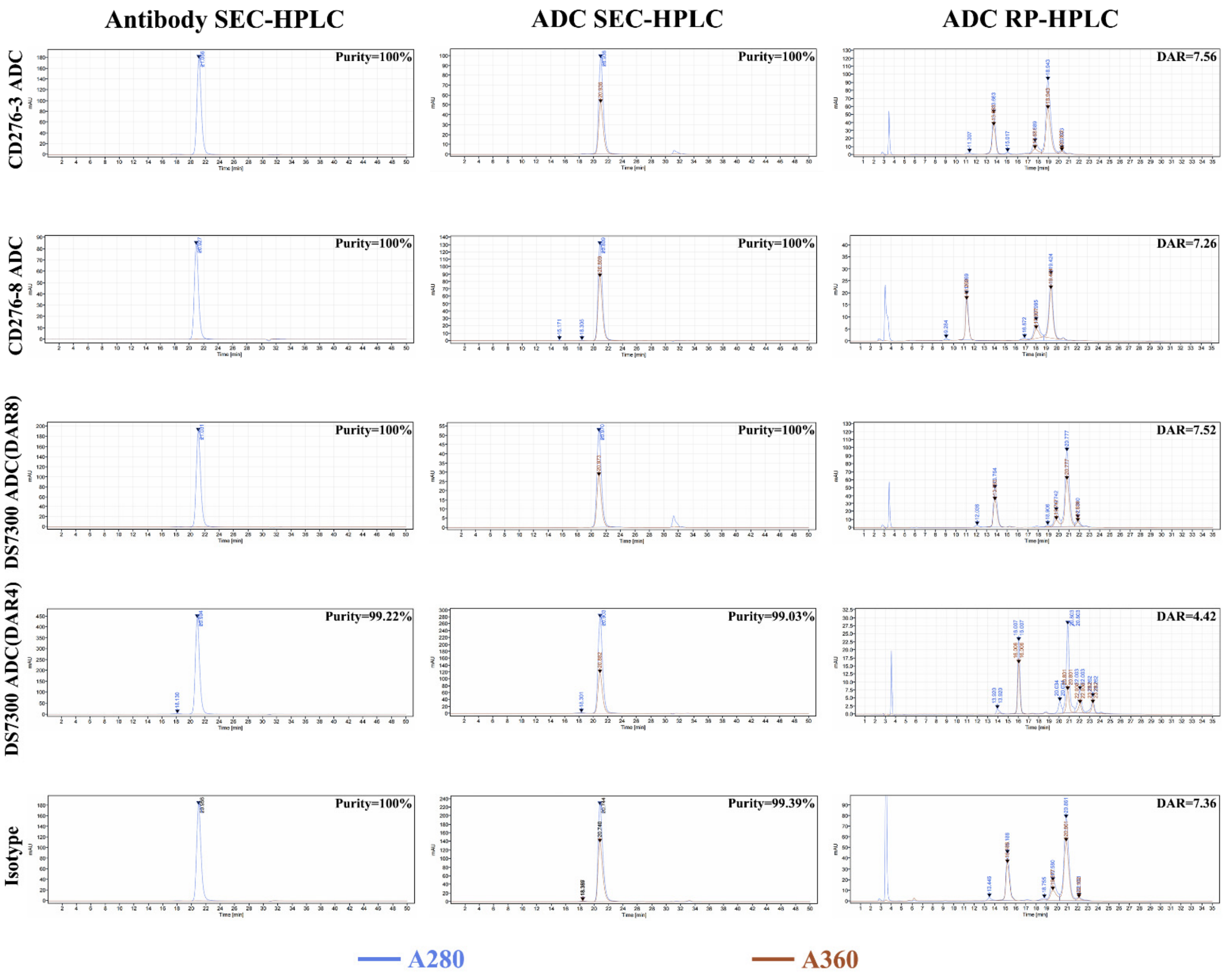

Figure S3. Characterization of CD276-3 ADC, CD276-8 ADC, DS7300 ADC (DAR8), DS7300 ADC (DAR4) and isotype ADC. The purities of CD276-3, CD276-8, DS7300 and isotype antibodies and ADCs were detected by SEC-HPLC. The DAR of each ADC was detected by RP-HPLC.

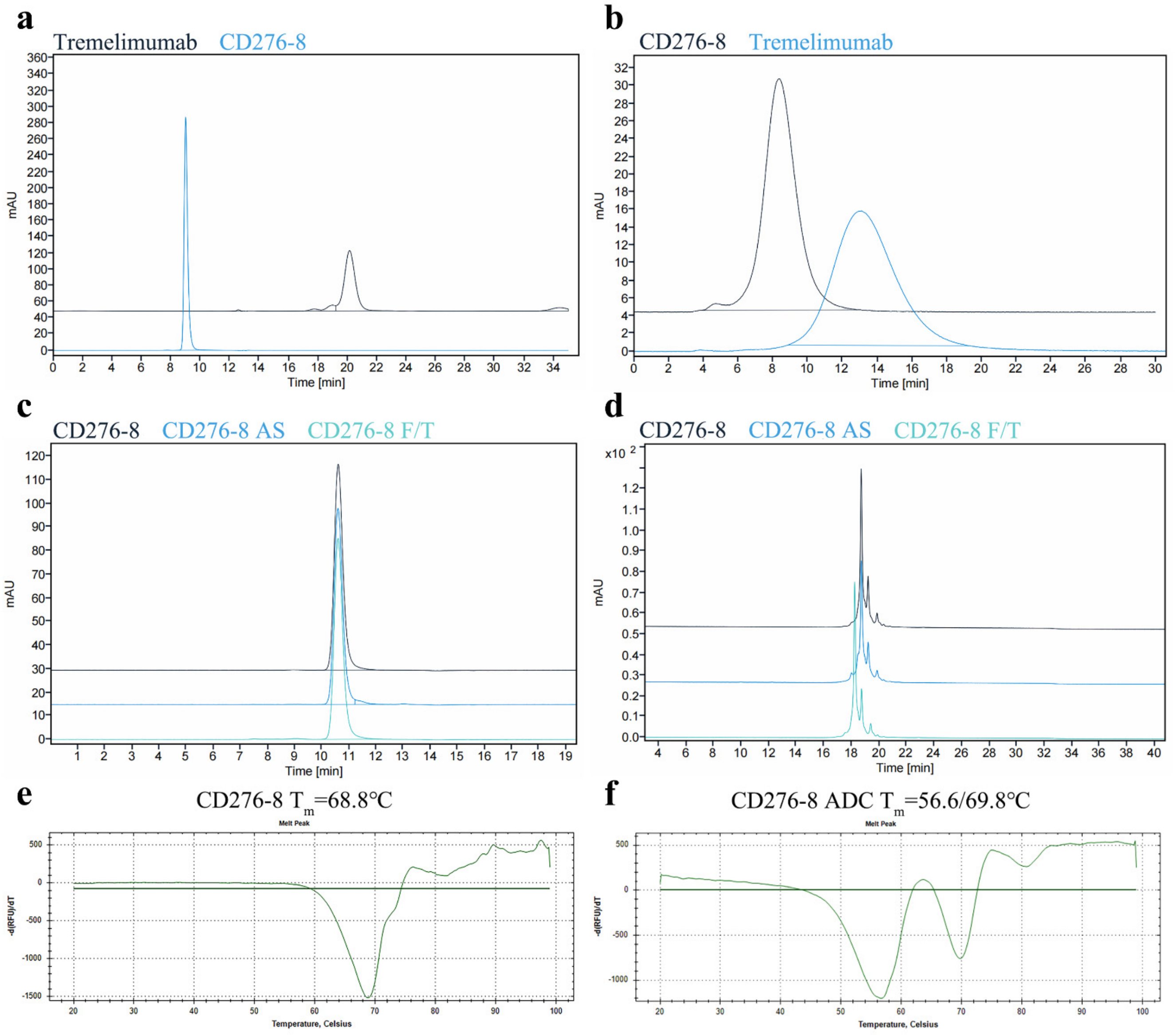

Figure S4. The developability of CD276-8 and thermal stability of CD276-8 and CD276-8 ADC. **(a)** Colloid stability of CD276-8 was determined by SMAC-HPLC compared with Tremelimumab. **(b)** Nonspecific interaction of CD276-8 was assessed by CIC-HPLC compared with Tremelimumab. **(c)** Purity of CD276-8 sample from AS or F/T study was assessed by SEC-HPLC. **(d)** Charge variants of CD276-8 sample from AS or F/T study was assessed by WCX-HPLC. **(e)** Thermal stability of CD276-8 was detected by DSF. **(f)** Thermal stability of CD276-8 ADC was detected by DSF.

Table S1. Summary of *in vitro* cytotoxicity (IC_{50}) of B7-H3 ADCs in various cell lines

Cell line	Group	IC_{50} (nM)	95% Confidence Interval (nM)	Span (%)	R^2
**Raji**	CD276-3 ADC	51.99	very wide	82.92	0.8925
	CD276-8 ADC	52.14	very wide	73.53	0.9179
	DS7300 ADC (DAR8)	55.13	very wide	77.15	0.9338
	Isotype ADC	61.31	42.94 - 1724	81.84	0.9323
**U251**	CD276-3 ADC	>200	very wide	Ambiguous	Ambiguous
	CD276-8 ADC	>200	very wide	Ambiguous	Ambiguous
	DS7300 ADC (DAR8)	>200	very wide	Ambiguous	Ambiguous
	Isotype ADC	>200	very wide	Ambiguous	Ambiguous
**A375**	CD276-3 ADC	23.91	19.34 - 30.18	87.06	0.9903
	CD276-8 ADC	38.67	33.24 - 45.44	92.54	0.9939
	DS7300 ADC (DAR8)	12.97	11.37 - 14.85	102.2	0.9960
	Isotype ADC	104.1	86.29 - 126.9	88.80	0.9870
**A431**	CD276-3 ADC	176.7	150.5 - 211.7	84.18	0.9850
	CD276-8 ADC	>200	very wide	78.07	0.9932
	DS7300 ADC (DAR8)	196.2	187.4 - 206.1	79.62	0.9985
	Isotype ADC	>200	very wide	95.93	0.9834
**MDA-MB-231**	CD276-3 ADC	>200	very wide	Ambiguous	Ambiguous
	CD276-8 ADC	>200	very wide	Ambiguous	Ambiguous
	DS7300 ADC (DAR8)	>200	very wide	Ambiguous	Ambiguous
	Isotype ADC	>200	very wide	Ambiguous	Ambiguous
**NCI-N87**	CD276-3 ADC	>200	very wide	Ambiguous	Ambiguous
	CD276-8 ADC	>200	very wide	Ambiguous	Ambiguous
	DS7300 ADC (DAR8)	>200	very wide	Ambiguous	Ambiguous
	Isotype ADC	>200	very wide	Ambiguous	Ambiguous
**HepG2**	CD276-3 ADC	>200	very wide	Ambiguous	Ambiguous
	CD276-8 ADC	>200	very wide	Ambiguous	Ambiguous
	DS7300 ADC (DAR8)	>200	very wide	Ambiguous	Ambiguous
	Isotype ADC	>200	very wide	Ambiguous	Ambiguous
**Huh7**	CD276-3 ADC	17.39	9.942 - 47.13	93.42	0.9776
	CD276-8 ADC	43.69	25.24 - 217.3	86.93	0.9681
	DS7300 ADC (DAR8)	5.265	3.001 - 14.93	87.28	0.9808
	Isotype ADC	>200	very wide	Ambiguous	Ambiguous
**HCC827**	CD276-3 ADC	21.73	14.18 - 34.11	85.94	0.9657
	CD276-8 ADC	15.04	9.279 - 26.40	94.44	0.9746
	DS7300 ADC (DAR8)	7.746	2.553 - 19.94	84.64	0.9428
	Isotype ADC	38.72	17.99 - 170.7	71.57	0.8732
**OVCAR3**	CD276-3 ADC	0.5388	0.4617 - 0.6382	80.62	0.9906
	CD276-8 ADC	2.802	2.456 - 3.225	85.94	0.9937
	DS7300 ADC (DAR8)	0.04758	0.04213 - 0.05353	85.79	0.9956
	Isotype ADC	20.73	17.67 - 24.14	91.62	0.9921
**PA-1**	CD276-3 ADC	1.897	1.636 - 2.163	106.5	0.9978
	CD276-8 ADC	12.43	10.91 - 14.19	98.95	0.9961
	DS7300 ADC (DAR8)	2.613	2.345 - 2.897	93.35	0.9974
	Isotype ADC	89.76	78.78 - 101.5	100.5	0.9928

Table S2. Summary of *in vivo* antitumor experiment conditions in different CDX mice models

Cell line	Cells per mouse	Therapy schedule	Group	Dose（mg/kg）
PA-1	9×10^{6}	Day 0	CD276-3 ADC	2.0
			CD276-8 ADC	2.0
			DS7300 ADC (DAR8)	2.0
			DS7300 ADC (DAR4)	2.0
			DS7300 ADC (DAR4)	4.0
			Isotype ADC	2.0
A375	5×10^{6}	Day 0	CD276-3 ADC	1.0
			CD276-8 ADC	1.0
			DS7300 ADC (DAR8)	1.0
			Isotype ADC	1.0
			CD276-3 ADC	3.0
			CD276-8 ADC	3.0
			DS7300 ADC (DAR8)	3.0
			Isotype ADC	3.0
Huh7	5×10^{6}	Day 0, Day 7	CD276-3 ADC	3.0
			CD276-8 ADC	3.0
			DS7300 ADC (DAR8)	3.0
			Isotype ADC	3.0
OVCAR3	5×10^{6}	Day 0	CD276-8 ADC	1.0
			Isotype ADC	1.0
